# Platelet-Derived Chemokine CXCL7 Dimer Preferentially Exists in the Glycosaminoglycan-Bound Form: Implications for Neutrophil–Platelet Crosstalk

**DOI:** 10.3389/fimmu.2017.01248

**Published:** 2017-10-02

**Authors:** Aaron J. Brown, Krishna Mohan Sepuru, Kirti V. Sawant, Krishna Rajarathnam

**Affiliations:** ^1^Department of Biochemistry and Molecular Biology, University of Texas Medical Branch, Galveston, TX, United States; ^2^Sealy Center for Structural Biology and Molecular Biophysics, University of Texas Medical Branch, Galveston, TX, United States; ^3^Department of Microbiology and Immunology, University of Texas Medical Branch, Galveston, TX, United States

**Keywords:** chemokine, glycosaminoglycan, heparin, NMR, CXCL7, NAP-2, dimer, CXCR2

## Abstract

Platelet-derived chemokine CXCL7 (also known as NAP-2) plays a crucial role in orchestrating neutrophil recruitment in response to vascular injury. CXCL7 exerts its function by activating the CXC chemokine receptor 2 (CXCR2) receptor and binding sulfated glycosaminoglycans (GAGs) that regulate receptor activity. CXCL7 exists as monomers, dimers, and tetramers, and previous studies have shown that the monomer dominates at lower and the tetramer at higher concentrations. These observations then raise the question: what, if any, is the role of the dimer? In this study, we make a compelling observation that the dimer is actually the favored form in the GAG-bound state. Further, we successfully characterized the structural basis of dimer binding to GAG heparin using solution nuclear magnetic resonance (NMR) spectroscopy. The chemical shift assignments were obtained by exploiting heparin binding-induced NMR spectral changes in the WT monomer and dimer and also using a disulfide-linked obligate dimer. We observe that the receptor interactions of the dimer are similar to the monomer and that heparin-bound dimer is occluded from receptor interactions. Cellular assays also show that the heparin-bound CXCL7 is impaired for CXCR2 activity. We conclude that the dimer–GAG interactions play an important role in neutrophil–platelet crosstalk, and that these interactions regulate gradient formation and the availability of the free monomer for CXCR2 activation and intrathrombus neutrophil migration to the injury site.

## Introduction

Akin to a well-equipped first aid kit used during emergencies, platelet granules contain hundreds of proteins that are rapidly released in response to vascular injury (1–4). These proteins mediate diverse functions from sealing wounds and sequestering the infection site to mobilizing leukocytes of the innate and adaptive arms of immunity. Chemokines constitute a critical component of this first aid kit, and it is now well established that they actively mediate aggregation and adhesion of platelets and recruit neutrophils through the thrombus body to the injury site (5–9). Recruited neutrophils mediate microbial killing, initiate tissue repair, and are essential for restoration of homeostasis. However, persistent neutrophil activation exacerbates the initial injury, resulting in various thrombus-related cardiovascular and inflammatory diseases (10–13).

CXCL7 was initially identified in platelet-derived releasates more than 25 years ago as a neutrophil-activating chemokine (NAC) (14–16). The importance of this finding remained unclear until a recent study provided unambiguous evidence for an *in vivo* CXCL7 chemotactic gradient in the thrombus body, and that it is indispensable for intrathrombus neutrophil migration (17). CXCL7 is essentially expressed only in platelets as an inactive precursor, with its active form generated *in situ* at the injury site (18, 19). CXCL7 is a member of a subset of seven chemokines characterized by their N-terminal ELR motif, which function as agonists for the CXC chemokine receptor 2 (CXCR2) receptor. These chemokines also share the properties of reversibly existing as monomers and dimers and interacting with glycosaminoglycans (GAGs).

Heparan sulfate (HS) is the predominant endothelial GAG, and is the glycan part of proteoglycans that span the membrane surface. HS also exists freely in the glycocalyx that dominates the luminal side of the endothelium. GAG interactions have been proposed to determine the nature and duration of chemokine gradients that play a fundamental role in regulating neutrophil trafficking.

CXCL7 exists as monomers, dimers, and tetramers (20). However, the dimer levels are low as the monomer dominates at lower and the tetramer dominates at higher concentrations. These observations raise the question—what, if any, is the role of the dimer? In this study, we make a striking observation that the dimer is actually the favored form in the GAG-bound state. However, structural characterization of the dimer and its GAG and receptor interactions is challenging considering native CXCL7 dimer is the minor species in the free form. Nuclear magnetic resonance (NMR) is ideally suited for such a task but presents its own challenge of requiring dimer chemical shift assignments. We accomplished this task by assigning chemical shifts of a disulfide-linked obligate dimer and by exploiting heparin binding-induced NMR spectral changes in the WT monomer and dimer. We successfully characterized the binding interactions of the native CXCL7 dimer to receptor N-domain and GAG heparin. These data indicated that the receptor interactions of the dimer are similar to the monomer and that GAG-bound dimer is occluded from receptor interactions. Functional studies also indicated that the heparin-bound CXCL7 is impaired for receptor activity.

On the basis of these data, we propose that the dimer actually plays a prominent role in neutrophil-platelet crosstalk through GAG interactions. Considering the local CXCL7 concentration can vary by orders of magnitude during active neutrophil recruitment, GAG-dimer interactions most likely regulate the levels of free monomer available for receptor activation. We further propose, in the context of platelet-neutrophil crosstalk, that the relative ratios of the monomer and dimer in the free and GAG-bound form could be critical for the repair process and to minimize collateral tissue damage and disease.

## Experimental Procedures

### Design, Expression, and Characterization of Trapped Dimer

A trapped CXCL7 dimer was designed by substituting a cysteine for Glu23 of the first β-strand that constitutes the two-fold symmetry axis. The protein was cloned, expressed, and purified as described previously (21). The gene corresponding to E23C CXCL7 was ligated into the pET 32Xa vector and expressed as a thioredoxin fusion protein with a His-tag. Proteins were expressed in the *E. coli* BL21 (DE3) strain either in ^15^N- or ^15^N/^13^C-enriched minimal medium. Transformed cells were grown to an A_600_ of 0.6, induced with 0.2 mM isopropyl β-d-thiogalactopyranoside, and grown for 16 h at 25°C. The protein-containing supernatant was loaded on to a Ni-NTA column and eluted with the same buffer as above, except containing 250 mM imidazole. Fractions containing the fusion protein were pooled and dialyzed against the cleavage buffer (20 mM Tris, 50 mM NaCl, 2 mM CaCl_2_, pH 7.4). The fusion tag was cleaved with Factor Xa, and the proteins were purified on reversed-phase high-pressure liquid chromatography (HPLC) using a gradient of acetonitrile in 0.1% heptafluorobutyric acid. The purity and molecular weight of the proteins were confirmed using analytical HPLC and matrix assisted laser desorption/ionization mass spectrometry respectively.

### Reagents

The recombinant CXCR2 N-terminal domain (N-domain) (residues 1–43) peptide was expressed using the same protocol as described for the trapped dimer. Heparin dp26 (degree of polymerization 26 and corresponds to a 26mer) oligosaccharide was purchased from Iduron (UK). According to the manufacturer, the oligosaccharides were purified using high resolution gel filtration chromatography, the main disaccharide unit is IdoA, 2S-GlcNS, 6S (~75%), show some variation in sulfation pattern, contain uronic acid at the non-reducing end, and a C4–C5 double bond as a result of the heparinase endolytic action.

### Chemical Shift Assignments of the CXCL7 Dimer

Nuclear magnetic resonance samples were prepared in a 50-mM phosphate buffer, pH 6.0, containing 1 mM 2,2-dimethyl-2-silapentansesulfonic acid (DSS), 1 mM sodium azide, and 10% D_2_O. NMR spectra were acquired on a Bruker Avance III 600 (with a QCI cryoprobe) or 800 MHz (with a TXI cryoprobe) spectrometer and processed and analyzed using either Bruker Topspin 3.2 or Sparky programs (22). Chemical shift assignments of the trapped dimer were determined at 30°C using a 400-µM sample. The ^1^H and ^15^N chemical shifts were assigned using 3D ^15^N-edited NOESY and TOCSY experiments with mixing times of 150 and 80 ms, respectively. The carbon chemical shifts assignments were obtained from HNCA and CBCACONH experiments at pH 6.0.

### NMR Titrations

Binding interactions of CXCR2 N-terminal domain and heparin dp26 were characterized using solution NMR spectroscopy. A series of ^1^H-^15^N HSQC spectra were collected until there were no chemical shift changes. In the case of CXCR2 N-domain, we titrated 320 µM CXCR2 N-domain to 77 µM WT CXCL7 in 50 mM phosphate buffer at pH 6.0 and 35°C. The final molar ratio of CXCL7:CXCR2 N-domain was 1:3.5. For GAG interactions, 2.5 mM heparin dp26 was titrated into a ~100 µM sample of either WT or trapped CXCL7 in 50 mM phosphate pH 6.0 at 35°C. The final molar ratio for CXCL7:dp26 was 1:4. For all titrations, chemical shift perturbations (CSPs) were calculated as a weighted average of changes in the ^1^H and ^15^N chemical shifts as described previously (23).

### Molecular Docking Using High Ambiguity-Driven Biomolecular DOCKing (HADDOCK)

Molecular docking of heparin to the CXCL7 dimer was carried out using the HADDOCK approach as described previously (24–27). The CXCL7 dimer structure consisted of chains A and B from the tetramer structure (PDB ID:1NAP) (28) and the NMR structure of heparin 14-mer (PDB ID:1HPN) (29) were used for docking. The AB-type dimer was selected based on analysis of NMR chemical shifts that revealed a CXC-type dimer. Residues that showed NMR CSP above a threshold were included as Ambiguous Interaction Restraints. The pair-wise “ligand interface RMSD matrix” over all structures was calculated and the final structures were clustered using an RMSD cutoff value of either 4 or 7 Å for one or two GAGs, respectively. The clusters were then prioritized using RMSD and “HADDOCK score” (weighted sum of a combination of energy terms).

### CXCR2 Activity

The CXCR2 activity was determined using a Ca^2+^ release assay as described previously (30). Stably expressing CXCR2-HL60 (CXCR2-HL60) cells were cultured in RPMI 1640 supplemented with antibiotics (Pen–Strep, Gibco) and 10% FBS (Sigma). Differentiation into the neutrophil lineage was carried out by subculturing CXCR2-HL60 cells every other day by using antibiotic-free media containing 1.25% DMSO for 6 days (31). Ca^2+^ levels were measured using a FlexStation III microplate reader using the Calcium 6 assay kit (FLIPR, Molecular Devices). On day 6, CXCR2-HL60 cells were plated at a concentration of 2 × 10^5^cells/well in a 96-well black plate (Costar). The cells were incubated with varying concentrations of CXCL7 WT or trapped dimer, and changes in fluorescence were measured every 5 s for up to 240 s at room temperature. Ca^2+^ release activity of heparin-bound CXCL7 was determined in a similar manner. 2 nM CXCL7 was mixed with different concentrations of heparin (Iduron, UK) and immediately added to dye-loaded cells. Heparin by itself did not induce any Ca^2+^ release. Statistical significance was determined using one-way ANOVA followed by Tukey’s *post hoc* analysis; *p < 0.05.

## Results

### CXCL7 Dimer Exists in the GAG-Bound State

**Figure 1:**
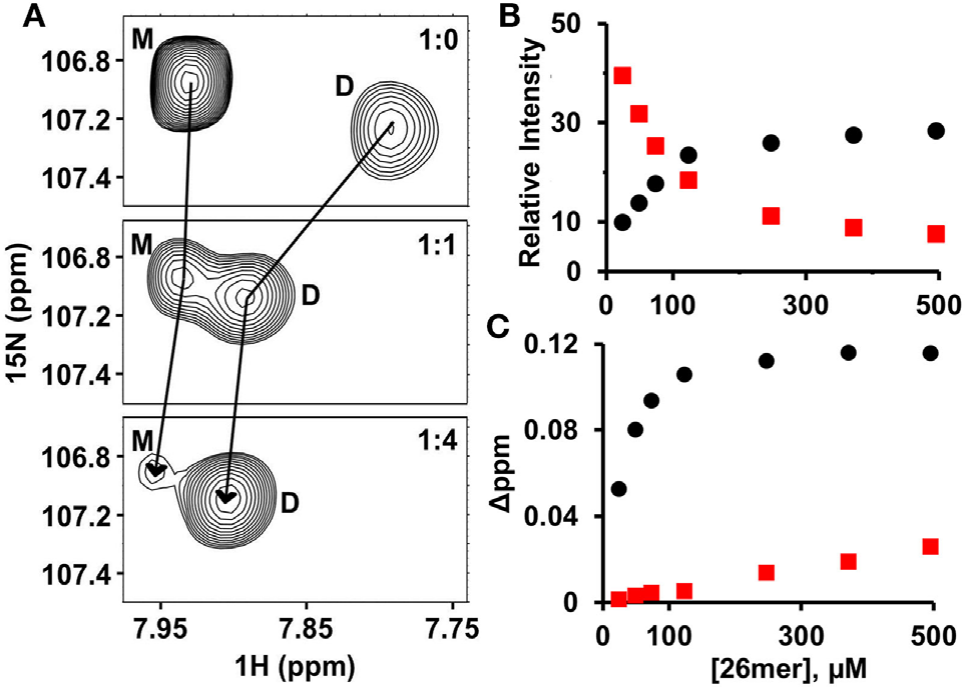
CXCL7 dimer is the high-affinity glycosaminoglycan ligand. **(A)** A section of the HSQC spectra showing heparin dp26 binding-induced transition from a predominantly monomeric to predominantly dimeric state. **(B)** Plot showing the relative change in peak intensity for monomer (red squares) and dimer (black circles). **(C)** Plot showing the change in chemical shifts for monomer (red squares) vs. dimer (black circles). Data are shown for residue G65.

Both HS and heparin share a repeating disaccharide unit composed of *N*-acetyl glucosamine and glucuronic acid. However, heparan sulfate (HS) has a modular structure with sulfated sequences (defined as NS domain) separated by non-sulfated regions containing acetylated sequences (defined as NA domain). Heparin is preferred for structural studies for the reason it is more uniformly sulfated, commercially available, and has been shown to capture endogenous interactions (32, 33). Various cellular, *ex vivo*, and biophysical studies for a wide variety of chemokines have shown that the dimer and higher order oligomers, compared to the monomer, bind GAGs such as heparan sulfate with higher affinity (24, 25, 34–38). In particular, recent NMR studies of GAG heparin binding to WT CXCL1, CXCL5, and CXCL8 have shown that the dimer is the high-affinity GAG ligand and that the differences in affinities are more pronounced for longer GAGs. We had previously observed that the affinity of the heparin octasaccharide for the monomer and dimer were essentially similar (20). With this in mind, we characterized the binding of heparin 26mer (dp26) to a 120-µM sample of WT CXCL7 at pH 6.0. Under these conditions, 70% of the protein exists as the monomer and the remaining as the dimer (20). In principle, four different species will exist in solution due to coupled equilibria—both monomer and dimer in the free and dp26-bound forms. The intensities of peaks are a direct reflection of the relative populations and binding constants. During the course of the titration, the peaks corresponding to the monomer disappear and the weak peaks corresponding to the dimer gain intensity, indicating that the dimer binds dp26 with much higher affinity (Figure 1A). In addition to peak intensity changes, CSP profiles for the dimer compared to the monomer also indicates that the dimer is the high-affinity ligand (Figures 1B,C).

### Chemical Shift Assignments of the CXCL7 Dimer

Knowledge of the CXCL7 dimer chemical shifts is essential to describe the molecular basis of receptor and GAG interactions. However, unlike for the CXCL7 monomer, dimer does not dominate at any solution condition making its chemical shift assignments challenging. To overcome this challenge, we used a multi-pronged strategy that included chemical shift assignments of a disulfide-linked trapped dimer, heparin binding-induced chemical shift changes in the trapped dimer, and heparin binding-induced intensity and chemical shift changes in the WT monomer and dimer. These collectively allowed assignments of 80% of the residues including all of the residues that showed perturbation on heparin binding.

A trapped dimer was designed by introducing a disulfide bond across the twofold symmetry axis of the dimer interface (39), which involved mutating the dimer interface residue E23 to cysteine. The formation of the disulfide-trapped dimer was confirmed using SDS-PAGE, mass spectrometry, and NMR spectroscopy. NMR Cβ chemical shift of the newly introduced cysteine (45.3 ppm) indicated it is in the disulfide bonded state (40) (Figure S1 in Supplementary Material). Backbone NH chemical shifts of the trapped dimer were assigned using a combination of ^15^N-edited NOESY, ^15^N-edited TOCSY, HNCA, and CBCACONH experiments.

The trapped dimer chemical shifts allowed assigning 35 native dimer residues in a straightforward manner as these residues had essentially identical nitrogen and amide chemical shifts. Assignments for another 13 residues were obtained by comparing heparin binding-induced NMR spectral changes in the WT and trapped dimer. In the WT titrations, weak dimer peaks become strong and monomer peaks become weak, which, combined with differential chemical shift changes from the monomer, allowed for unambiguous tracking of the WT dimer chemical shifts and comparison to known trapped dimer chemical shifts. Finally, we assigned six residues on the basis that the intensity of these peaks did not change during the WT titration, indicating the dimer and monomer chemical shifts of these residues are identical. These residues were, therefore, assigned on the basis of known monomer chemical shifts. We could not unambiguously assign the remaining 13 residues, but lack of this knowledge was not limiting as these residues showed minimal to no perturbation in both receptor N-domain and GAG-binding experiments.

### CXCL7 Dimer–Heparin Interactions

**Figure 2:**
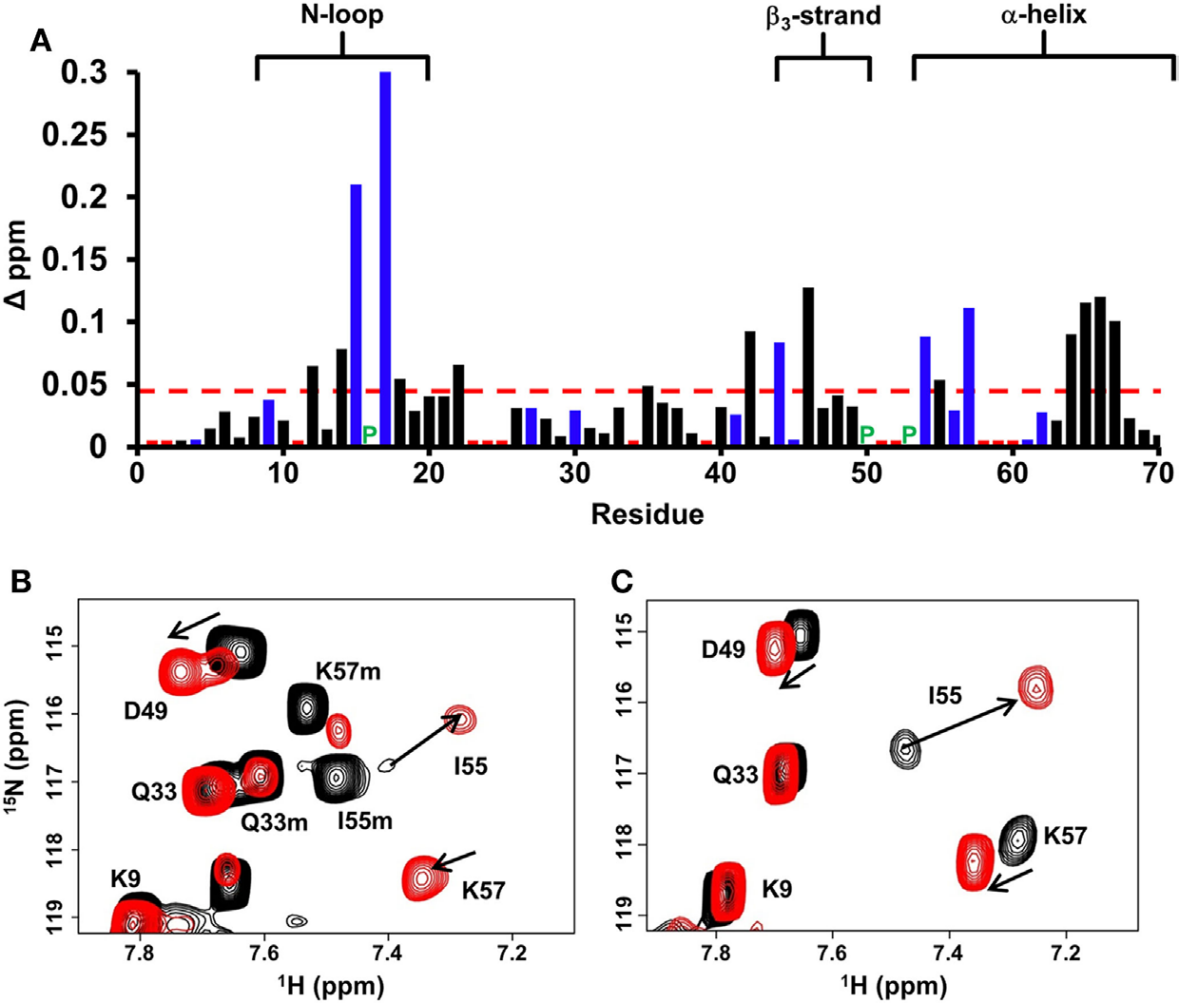
CXCL7 dimer binding to heparin dp26. **(A)** Histogram plot of binding-induced chemical shift changes in CXCL7 dimer as a function of amino acid sequence. Residues that show chemical shift perturbation above the threshold (dashed line) are considered involved in binding. Basic residues Arg, Lys, and His are shown in blue. None of the unassigned residues (shown in red) showed perturbation. Proline is represented by a green “P.” **(B)** Portion of the ^1^H–^15^N HSQC spectra showing the overlay of native CXCL7 in the free (black) and heparin dp26-bound form at a 1:4 M ratio (red). **(C)** Portion of the ^1^H–^15^N HSQC spectra showing the overlay of the trapped CXCL7 dimer in the free (black) and dp26-bound form at a 1:4 M ratio (red). In both spectra, the dimer peaks are labeled for reference. Monomer peaks are indicated with an “m.” Arrows indicate the direction of peak movement.

Chemical shift perturbation profile of dp26 binding to the WT dimer revealed a contiguous surface consisting of residues from the N-loop, β_3_-strand, and α-helix. Most importantly, large chemical shift changes were observed for basic residues H15, K17, R44, R54, and K57 (Figure 2A). In the case of trapped dimer, the same residues not only showed similar perturbations, but the magnitude and direction of the perturbations were also essentially similar (Figures 2B,C).

To gain insight into the binding geometry, we performed four independent HADDOCK-based calculations to ensure that both a 1:1 and 1:2 stoichiometry were covered and to avoid any inherent bias in the docking process. In run I, restraints were given between one GAG and to only one monomer of the dimer. In run II, restraints were given between one GAG and both monomers of the dimer. In run III, restraints were given from each of two GAGs to either monomer of the dimer. Finally, in run IV, restraints were given from each of two GAGs to both monomers of the dimer.

**Figure 3:**
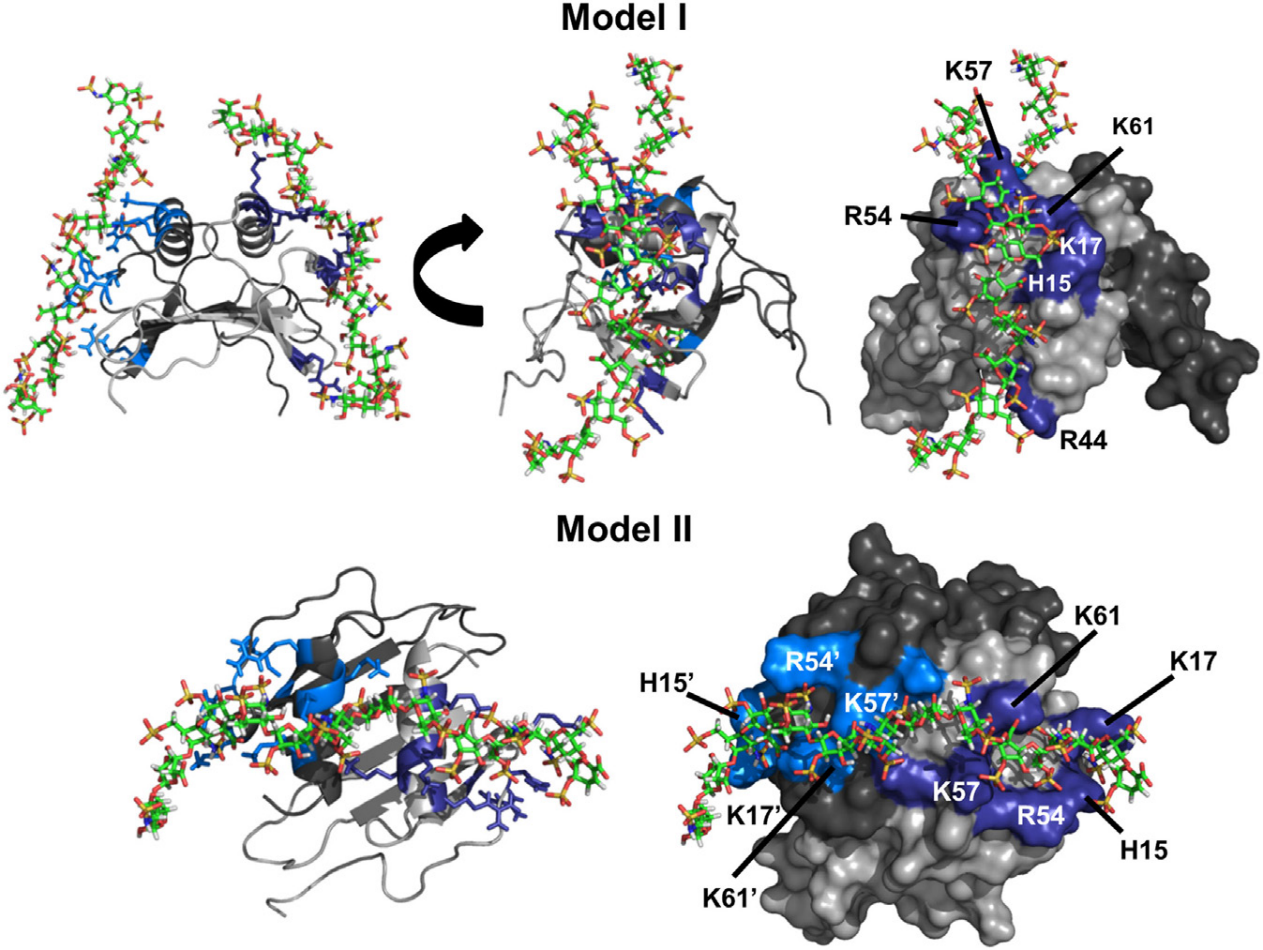
CXCL7 dimer heparin-binding models. Schematic showing the two primary models of heparin binding to the CXCL7 dimer. Each monomer of the dimer is shaded differently for clarity. Heparin-binding residues Arg, Lys, and His are highlighted in blue and labeled. Model-I depicts binding of two heparin chains to each monomer of the dimer, and two different views are shown to highlight the binding geometry. Model-II depicts binding of a single heparin across the dimer interface and is shown from a top-down view.

Run I essentially resulted in a single geometry that could be divided into two major clusters. In one cluster, all residues implicated from NMR studies were involved in binding, whereas the second cluster was missing interactions from R44. Comparison of the two clusters revealed that H15, K17, R54, K57, and K61 function as a core domain and R44 functions as a peripheral residue, suggesting that interactions with R44 are more transient. Runs III and IV resulted in a single geometry with one GAG binding each monomer of the dimer with geometries observed for run I. We define this binding geometry as Model-I (Figure 3). Interestingly, run II resulted in a different geometry (defined as Model-II) in which a single GAG spans both monomers of the dimer, and in this geometry, all residues except R44 from both monomers mediate binding.

### CXCL7 Dimer–CXCR2 Interactions

**Figure 4:**
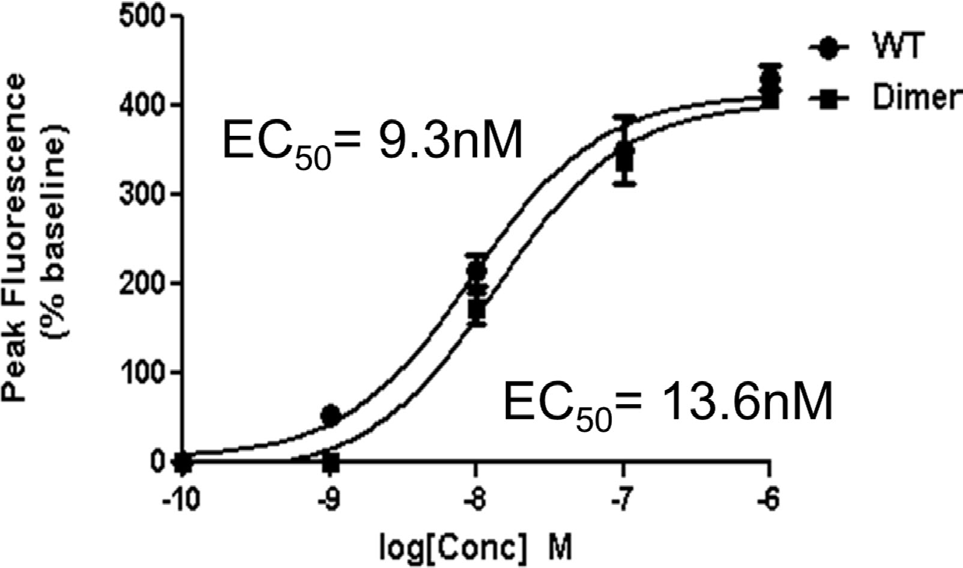
CXCR2 activity of the trapped dimer. A plot showing Ca^2+^ release activity profiles of CXCL7 WT and trapped dimer. Measurements were carried out using differentiated HL60 cells expressing CXCR2. The EC_50_ values indicate that the activity of the dimer is similar to the WT. The plot shown is a representative of three independent experiments where data were collected in quadruplicate for each treatment.

Though our studies indicate that the dimer preferentially exists in the GAG-bound form, knowledge of its receptor activity and the structural basis for these interactions is necessary to fully understand the role of the dimer in the context of *in vivo* function. We characterized receptor activity of the CXCL7 dimer by measuring Ca^2+^ release using HL60 cells stably transfected with the CXCR2 receptor (30). The trapped dimer was as potent as the WT indicating that the activities of the monomer and dimer are similar (Figure 4). Previous studies using a trapped dimer for related CXCR2 agonists CXCL1 and CXCL8 have also shown that the dimer could be as active as the monomer (30, 41).

It is now well established that receptor activation involves binding two distinct receptor sites. The initial binding involves the receptor N-terminal domain (defined as Site-I) and subsequent binding to a site involving extracellular loops and transmembrane domain (defined as Site-II). The structural basis for Site-I interactions can be characterized by using N-domain peptides (42–44). Site-I interactions of the CXCL7 monomer binding to the CXCR2 N-domain peptide have been characterized using solution NMR spectroscopy (20). We use the same divide and conquer approach for characterizing dimer interactions.

**Figure 5:**
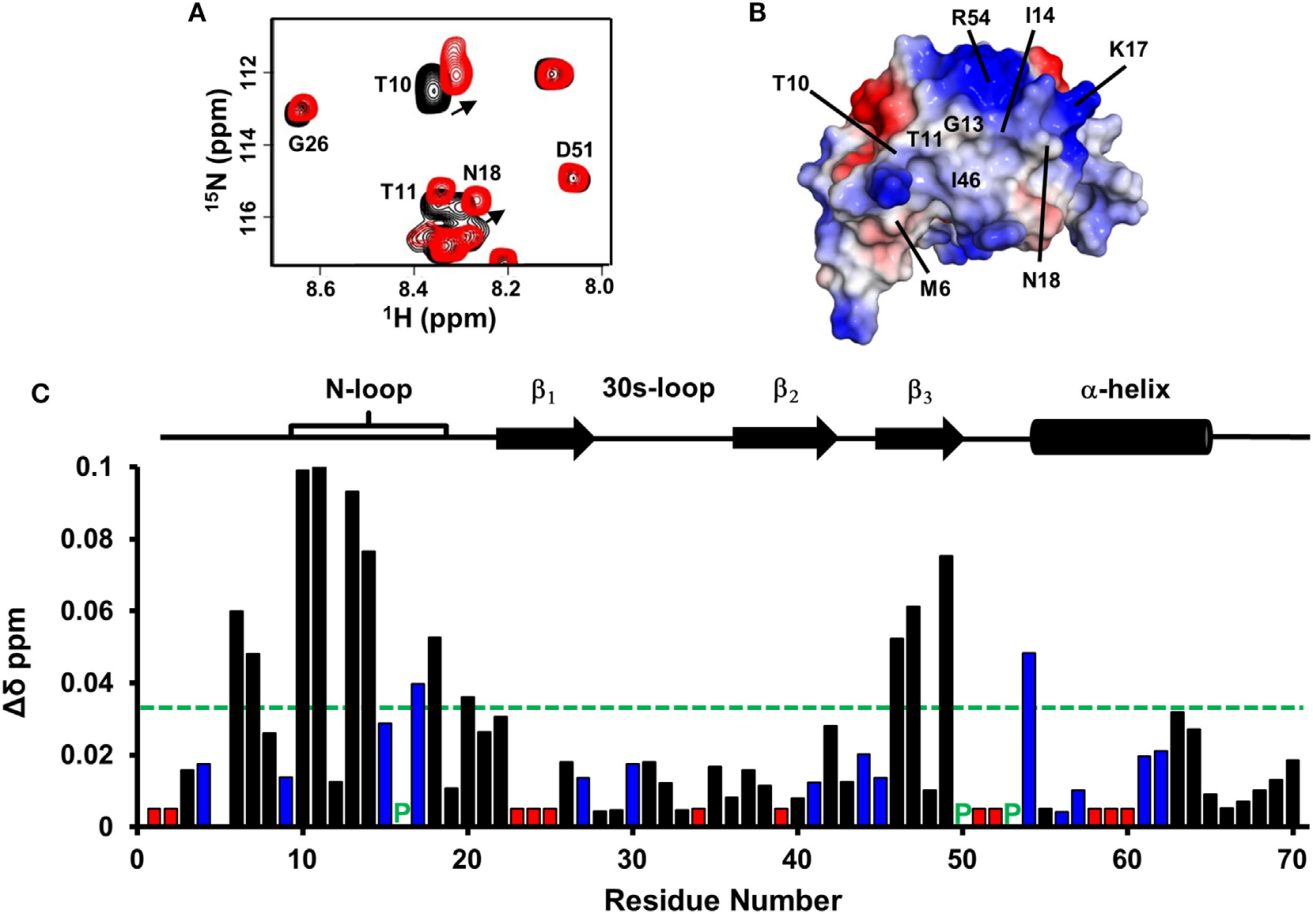
CXCL7 dimer binding to the CXCR2 N-domain. **(A)** Portion of the 2D HSQC spectrum showing the overlay of CXCL7 in the free (black) and in the presence of CXCR2 N-domain at a 1:3.5 M ratio (red). A subset of residues that show binding-induced chemical shift changes are labeled and arrows indicate direction of the peak movement. **(B)** Structure of the CXCL7 dimer highlighting the CXCR2 N-domain binding pocket. Electrostatic surface potential reveals a hydrophobic patch surrounded by basic residues. Note that these residues are located away from the dimer interface. **(C)** Histogram plot of binding-induced chemical shift changes in the CXCL7 dimer as a function of amino acid sequence. Basic residues are shown in blue, and unassigned residues are shown in red. Prolines are indicated by a green “P.” Residues that show chemical shift perturbation above the threshold (dashed line) are considered involved in binding. Secondary structural elements are given for reference.

Under the experimental conditions, CXCL7 exists as ~70% monomer and ~30% dimer. Armed with previously assigned monomer and dimer chemical shifts from our current study, we could simultaneously track binding-induced CSP for both dimer and monomer residues (20). We observe large CSP for residues M6, C7, T10, T11, G13, I14, K17, N18, I46, C47, D49, and R54 (Figures 5A,C). These residues constitute a hydrophobic pocket flanked by positively charged side chains involving the N-loop and β_3_-strand residues (Figure 5B). The extent of CSP and relative peak intensities for dimer and monomer were similar, indicating Site-I interactions of monomer and dimer are essentially the same.

### GAG-Bound Dimer Cannot Bind the Receptor

**Figure 6:**
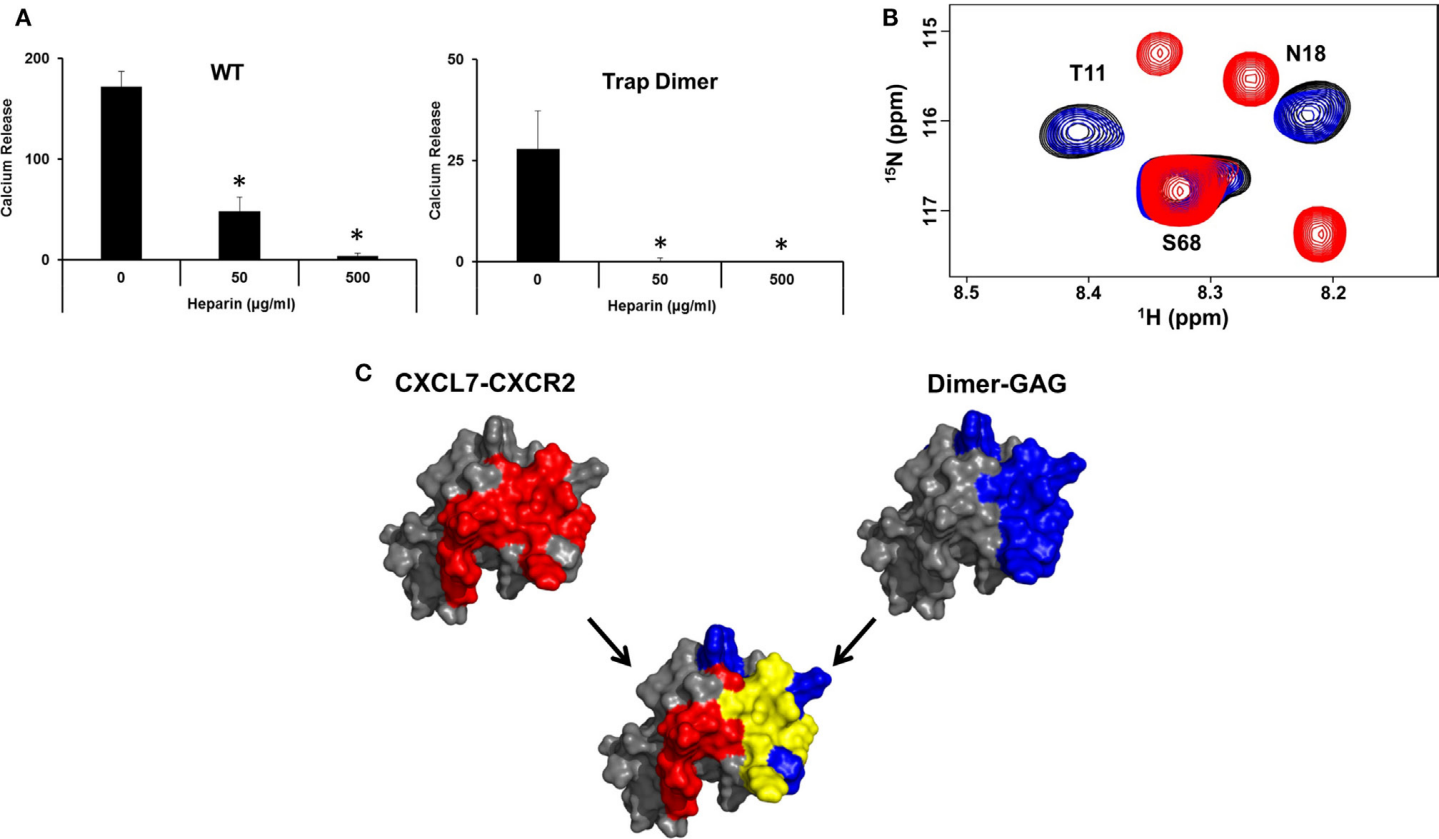
Overlap between heparin and CXCR2 binding domains. **(A)** Ca^2+^ release activity of 2 nM CXCL7 WT and trapped dimer in the presence of 50 and 500 µg/ml heparin. Data presented are the means ± SE from three independent experiments, with each treatment performed in quadruplicate. **(B)** Section of the ^1^H–^15^N HSQC spectrum showing peaks corresponding to the CXCL7:dp26 complex (black), the CXCL7:CXCR2 N-domain complex (red), and the CXCL7:dp26 complex upon titrating CXCR2 N-domain peptide up to a 1:7 M ratio (blue). **(C)** A schematic showing the CXCR2 binding domain (red), heparin-binding domain (blue), and the overlap between the two domains (yellow) for the CXCL7 dimer. The monomer structure is used for clarity. Extensive overlap between the heparin and receptor domains indicates that heparin-bound CXCL7 cannot bind the receptor.

A central question in determining the *in vivo* role of dimer is how GAG binding relates to receptor activation. We observe that heparin-bound CXCL7 is impaired for CXCR2 activity (Figure 6A). NMR studies also indicated that the heparin-bound CXCL7 is unable to bind the CXCR2 N-domain peptide (Figure 6B). Independent of any binding models, we observe that there is considerable overlap between the heparin and CXCR2 binding domains, providing a structural basis as to why heparin-bound CXCL7 is unable to bind the receptor (Figure 6C).

## Discussion

In the event of vascular injury, crosstalk between activated platelets and neutrophils is essential for initiating repair and successful restoration of homeostasis. However, any dysregulation in this process also aggravates the course of various thrombus-related cardiovascular and inflammatory diseases. Chemokine CXCL7, released by activated platelets, plays a prominent role in recruiting neutrophils to the injury site. CXCL7 elicits its function by binding to GAGs and the CXCR2 receptor. CXCL7 is unique as it is the only NAC that is almost exclusively present only in platelets, and it is also unusual for the reason that it forms a weak dimer. Therefore, the functional relevance of the dimeric state, if any, has not been addressed. Our current studies provide compelling evidence that the dimer is the favored form in the GAG-bound state.

We characterized how the native CXCL7 dimer binds to GAG heparin using solution NMR spectroscopy and HADDOCK modeling, and observed two distinct binding models. Model-I involved a stoichiometry of two GAGs per dimer and Model-II involved a stoichiometry of one GAG per dimer. Of the two models, we propose that the two GAG-binding model could be the preferred mode of interaction as it satisfies all binding interactions as inferred from the NMR studies. A stoichiometry of two GAGs binding per dimer has also been proposed for related NACs, though the binding geometries are different (24, 25, 38).

Previously, we had characterized heparin dp8 binding to the CXCL7 monomer (20). Large CSPs were observed for residues H15, K17, R44, R54, and K57 as seen for the CXCL7 dimer. In addition, perturbations were also observed for basic residues K9, K45, and K56 in the monomer. Absence of perturbations for these residues in the dimer indicates that the topology of the basic residues in the context of dimeric structure results in a more selective binding geometry. Another interesting difference is in the C-terminal helical residues A64 to D70. Whereas these residues were perturbed in the monomer, only residues A64–A67 are perturbed in the dimer. These differences may be explained by a more defined helix, or more restricted dynamics in the dimer, and therefore, GAG-binding results in less structural changes and consequently less or no chemical shift changes.

**Figure 7:**
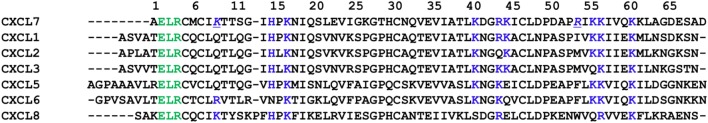
Sequence alignment of neutrophil-activating chemokines. Conserved “ELR” motif is shown in green, and potential heparin-binding residues from CXCL7 are shown in blue. Heparin-binding residue R54 unique to CXCL7 is underlined and italicized.

Comparison of heparin-binding residues between CXCL7 and other NACs reveals both highly conserved and CXCL7-specific interactions. Highly conserved residues include H15, K17, K57, and K61, and these residues mediate heparin binding in all NACs studied to date (Figure 7). R54 is unique to CXCL7 and serves as a core-binding residue. Further, the heparin-binding geometry in the CXCL7 dimer is different compared to other NACs (24, 25, 38, 45). Our data suggest heparin binds CXCL7 perpendicular to the helices across the N-loop spanning from the helix to the third β-strand. The binding interface for the CXCL1 dimer is distinctly different, with two heparins binding across the dimer interface on opposite faces of the protein (24). The binding geometry in CXCL5 is most similar to that of the CXCL7 dimer, except for differences due to CXCL7 R54 directing heparin toward the N-terminal end of the helix and additional C-terminal helical residues mediating CXCL5 interactions (38). These observations indicate that conserved and specific residues in the context of structure determine geometries that could not have been predicted from sequence alignment alone. These studies also speak to the rich diversity in GAG interactions for related proteins that most likely play a role in fine-tuning chemokine-specific neutrophil function.

NMR studies reveal that the N-loop and adjacent β-strand residues of the CXCL7 dimer mediate binding to the CXCR2 N-domain. The binding mode and the nature of these interactions are similar to that observed for other CXCR2-activating chemokines CXCL1, CXCL5, and CXCL8 (24, 38, 44). Independent of the binding geometry and stoichiometry, comparison of the heparin and receptor binding domains reveals a number of residues play dual roles by binding both, indicating heparin-bound CXCL7 dimer is precluded from interacting and activating the receptor. This is corroborated by the observation that heparin-bound CXCL7 is impaired for CXCR2 activity, and that CXCR2 N-domain peptide is unable to bind the heparin-bound CXCL7 dimer.

The importance of the chemokine monomer-dimer equilibrium for neutrophil trafficking has been demonstrated using animal models (32, 33, 46, 47). During neutrophil recruitment into the thrombus, CXCL7 is released at high concentrations from α-granules of activated platelets (48). This results in CXCL7 being present over a large concentration range as a function of space and time, wherein CXCL7 can exist as monomers and dimers in the free and/or GAG-bound forms. GAGs exist on the endothelial surface, in the glycocalyx that dominates the luminal side of the endothelium, and platelet granules also release proteoglycans. Therefore, circulating neutrophils will encounter free and GAG-bound CXCL7 gradients that will lead to their arrest and adhesion to the endothelium. Our data indicate that the monomer exists predominantly in the free and the dimer in the GAG-bound form. Our observation for the dimer is unexpected and interesting considering free dimer was a minor species at all concentrations. Therefore, GAG binding overcomes interactions that disfavor dimerization in the free form, and knowledge of the structure of the heparin-dimer complex is necessary to understand the structural basis for complex formation. In the context of *in vivo* function, tetramers will also play a role. At this time, nothing is known regarding tetramer–GAG interactions. Under our experimental conditions, we did not see any evidence for a tetramer in the heparin-bound form suggesting that the heparin affinity of the tetramer is similar, and definitely not significantly higher, to that of the dimer. Our future studies will address the relationship between dimer-tetramer equilibrium and GAG interactions. Recent studies have shown CXCL7 forms heterodimers with other platelet-derived chemokines (6–8, 49, 50), and that the heterodimer binds GAG with high affinity suggesting GAG interactions of both homodimers and heterodimers could regulate function.

In summary, we conclude that the dimeric form plays an important role in neutrophil–platelet crosstalk as it is the dominant GAG-bound form, and GAG interactions determine the levels of free CXCL7 available for receptor activation and influence the makeup of gradients for intrathrombus neutrophil migration. The ability of CXCL7 to exist as a monomer and dimer and in the free and GAG-bound states could be critical in the context of platelet–neutrophil communication to maximize repair and minimize collateral tissue damage and disease.

## Author Contributions

KR and AB designed the research and wrote the paper. AB, KMS, and KVS performed the experiments and analyzed the data. All authors reviewed the results and approved the final version of the manuscript.

## Conflict of Interest Statement

The authors declare that the research was conducted in the absence of any commercial or financial relationships that could be construed as a potential conflict of interest.

## Abbreviations

CXCL, CXC ligand; CXCR2, CXC chemokine receptor 2; CXCL7/NAP-2, neutrophil-activating peptide 2; GAG, glycosaminoglycan; N-domain, N-terminal domain; GPCR, G-protein coupled receptor; NAC, neutrophil-activating chemokine; NMR, nuclear magnetic resonance; HSQC, heteronuclear single quantum coherence; CSP, chemical shift perturbation; NOE, nuclear Overhauser effect; AIR, ambiguous interaction restraints.
