# Supplementary material for: Platelet-Derived Chemokine CXCL7 Dimer Preferentially Exists in the Glycosaminoglycan-Bound Form: Implications for Neutrophil–Platelet Crosstalk
Source: Front Immunol. 2017 Oct 2;8:1248. doi: 10.3389/fimmu.2017.01248 (PMC5630695; doi:10.3389/fimmu.2017.01248)
Supplement: Supplementary file 1 [file Data_Sheet_1.docx]

**Supplementary Material**

**Platelet-derived chemokine CXCL7 dimer preferentially exists in the glycosaminoglycan-bound form: Implications for neutrophil-platelet crosstalk**

Aaron J. Brown^1,2^, Krishna Mohan Sepuru^1,2^, Kirti V. Sawant^1^, and Krishna Rajarathnam^1,2,3*^

^1^Department of Biochemistry and Molecular Biology, University of Texas Medical Branch, Galveston, TX, USA. ^2^Sealy Center for Structural Biology and Molecular Biophysics, University of Texas Medical Branch, Galveston, TX, USA. ^3^Department of Microbiology and Immunology, University of Texas Medical Branch, Galveston, TX, USA.


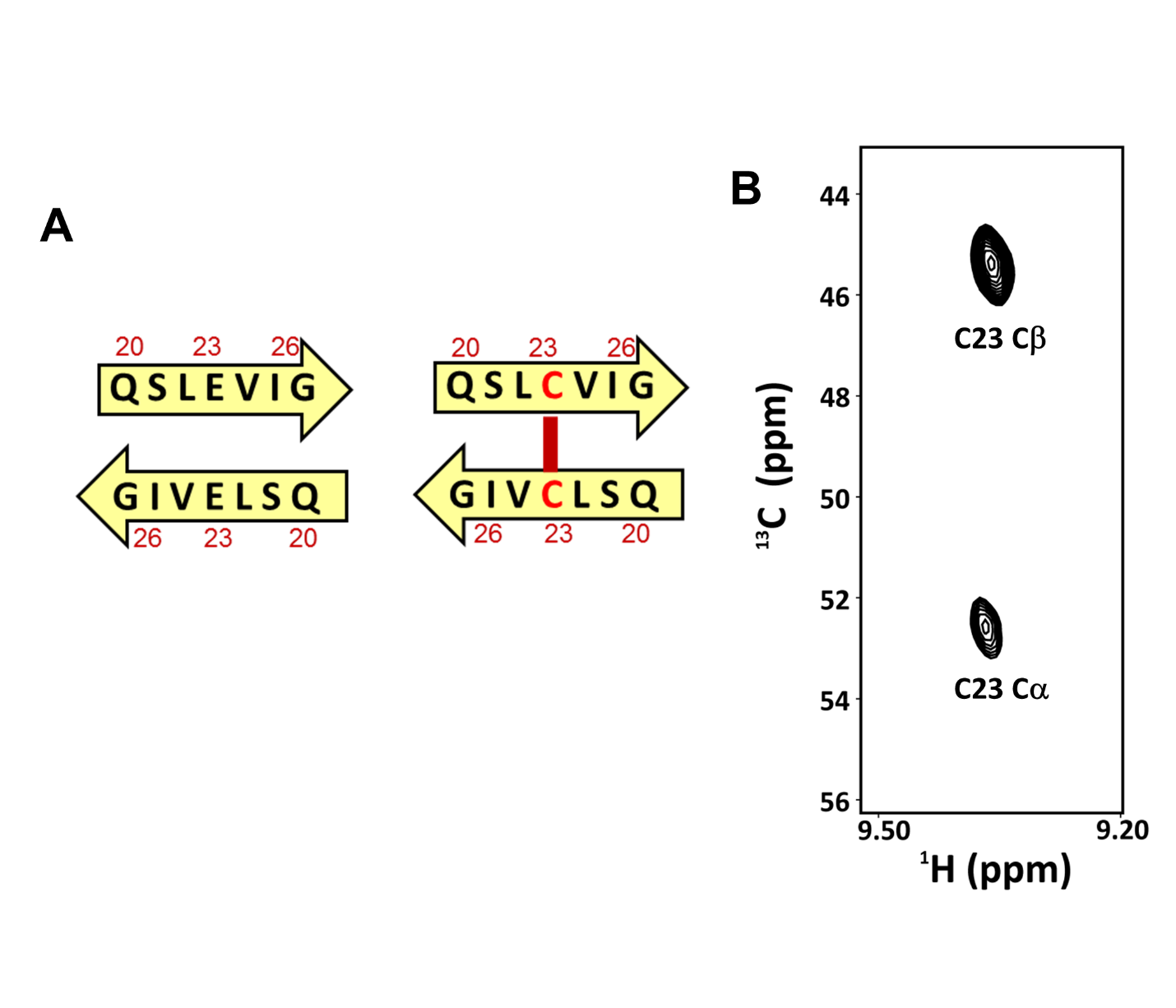


**Supplementary Figure 1. (A**) A schematic showing the design of a trapped dimer. Dimer interface residue Glu23 is mutated to a cysteine. (**B**). A strip plot showing C23 carbon chemical shifts. Cβ chemical shift of 45.3 indicates C23 is in the disulfide-bonded form (40).
